# Calcium Alginate-Neusilin US2 Nanocomposite Microbeads for Oral Sustained Drug Delivery of Poor Water Soluble Drug Aceclofenac Sodium

**DOI:** 10.1155/2015/826981

**Published:** 2015-02-23

**Authors:** Manjanna Kolammanahalli Mallappa, Rajesh Kesarla, Shivakumar Banakar

**Affiliations:** ^1^Department of Pharmaceutics, TVM College of Pharmacy, Bellary, Karnataka, India; ^2^Department of Pharmaceutics, Parul Institute of Pharmacy, Limda, Gujarat, India; ^3^Department of Pharmaceutical Chemistry, B.L.D.E College of Pharmacy, Bijapur, Karnataka, India

## Abstract

The aim of the present study was to formulate and investigate the calcium alginate- (CA-) Neusilin US2 nanocomposite microbeads containing preconcentrate of aceclofenac sodium (ACF-Na) liquid microemulsion (L-ME) for enhancement of oral bioavailability. The preconcentrate L-ME is prepared by using Labrafac PG, Labrasol, and Span 80 as oil, surfactant, and cosurfactant, respectively. The solid CA nanocomposite microbeads of L-ME prepared by microemulsification internal gelation technique using sodium alginate (SA) gelling agent, Neusilin US2 as adsorbent, and calcium chloride as crosslinking agent. L-ME has good thermodynamic stability; globule size was found to be 32.4 nm with polydispersity index 0.219 and −6.32 mV zeta potential. No significant interactions of excipients, drug in the formulations observed by FT-IR, DSC and XPRD. The concentration of SA and Neusilin US2 influences the flow properties, mean particle size, mechanical strength, drug entrapment efficiency, and percentage of drug release. All the formulations show minimum drug release in simulated gastric fluid (SGF) pH 1.2 for initial 2 h, maximum drug release in pH 6.8 phosphate buffer solution (PBS) at 6 h, followed by sustaining in simulated intestinal fluid (SIF) of pH 7.4 up to 12 h. The interaction of SA with Neusilin US2 creates a thick thixotropic gel network structure which acts as barrier to control the release of drug in the alkaline pH environment. Neusilin US2 is a novel filler used to convert L-ME into solid nanocomposite microbeads to enhance dissolution rate of poor water soluble drugs sustaining the drug release for prolonged period of time.

## 1. Introduction

Aceclofenac sodium (ACF-Na) is a nonsteroidal anti-inflammatory drug (NSAID) used extensively in the treatment of rheumatoid arthritis, osteoarthritis, and ankylosing spondylitis. It is rapidly and completely absorbed after oral administration, the biological half-life is 1.8–3.5 h, and dosing frequency is 2-3 times daily with dose range 100–200 mg [1]. However, ACF-Na is a water insoluble lipophilic drug coming under biopharmaceutical classification system (BCS) class II (low solubility and high permeability) which suffers limited oral bioavailability, high intra- and intersubject variability and lack of dose related adverse effects [2]. In recent years, liquid microemulsifying system (L-MES) is being investigated as potential new colloidal carrier for lipophilic drugs and has excellent thermodynamic stability, high drug solubilization capacity, nanosize (<200 nm), ease of scale-up and manufacturing, long shelf life, ability to improve dissolution rate and lymphatic transport of hydrophobic drugs, improved oral bioavailability, and protection against enzymatic hydrolysis [3]. Conventionally, L-MES is filled in hard or soft gelatin capsules for ease of administration. However, there exist a few limitations associated with this delivery system, including stability, manufacturing methods, leaking, leaching of components from the capsule shell, interaction of the fill with the capsule shell, and storage temperature [4]. To minimize the above-mentioned issues with L-MES, there is a growing trend to formulate solid microemulsifying system (S-MES) by adsorbing onto suitable carriers like lactose, natural gums, microcrystalline cellulose, and silicates to obtain dry powders [5]. Many literature reports conclude blending liquid systems with selected powder excipients to produce free flowing by using spray drying, solvent evaporation, and extrusion spheronization techniques [6]. But, in all these studies, to obtain solids with suitable processing properties, the required ratio of solidifying excipients to L-MES was very high and it seems to be practically infeasible for drugs having limited solubility in oil phase showing less drug content uniformity. Moreover, the powder formulations are primarily designed for filling into hard gelatin capsules; there is a limitation of how much powders may be filled into a capsule, especially by considering that most of the silicates have low bulk densities [7]. Therefore, better alternative for delivering L-MES can be modified into CA spheres or nanosized particles and easily filled in hard gelatin capsules and they readily form microemulsion gel layer due to swelling nature of sodium alginate which acts as barrier to diffuse the dissolved drug into bulk of the solution in a sustained manner [8]. Recently, microencapsulation has been employed to convert L-MES into solid spheres using dextran 40 as a water soluble solid carrier by spray drying method [9]. Microencapsulation is well accepted technique for development of homogeneous, monolithic particles in the range of about 0.1–1000 *μ*m and is employed to sustain the drug release [10]. Sodium alginate (SA) is a natural polysaccharide found in all species of brown algae and certain species of bacteria. It is a linear polymer of *β* (1–4) mannuronic acid (M) and *α* (1–4) guluronic acid (G) residue in varying proportions and arrangements [11]. SA can be ionically cross-linked with various types of cations like Ca^2+^, Ba^2+^, and Al^3+^ to form a viscous gel [12]. Among all that calcium ions interacting with alginates even small concentrations will produce very viscous gel compared to alginate alone. The gelation of alginic acid forms high viscous acid gel by intermolecular binding with calcium ion. During the cross-linking process, the water molecules will entrap inside the polymeric gel and are free to migrate throughout the gel layer [13]. This phenomenon has been applied to the preparation of CA beads for use as a drug delivery system, by dropping the drug-containing SA dispersion into a calcium chloride bath. CA microbeads have the advantages of being unable to reswell in acidic environment, whereas they easily reswell in an alkaline environment. So acid sensitive drugs incorporated into the CA beads would be protected from gastric juice [14].

Neusilin US2 is a synthetic, amorphous form of magnesium aluminometasilicate. It is a multifunctional excipient that can be used in both direct compression and wet granulation of solid dosage forms. It has good flow and compressibility properties and high specific surface area and adsorbs high loads of oils or water and can be mechanically compacted into high quality solid dosage forms [15]. Several literature reports explain that Neusilin US2 is a very good filler to convert the L-MES into solid microemulsifying drug delivery system (SMEDDS) [16].

The objectives of the present study were therefore (1) to develop ACF-Na microemulsion using the nonionic Labrasol (as surfactant HLB 16), Span 80 (as cosurfactant, HLB 4.6), and Labrafac PG, a lipophilic liquid, and water. Pseudoternary phase diagrams were constructed to find out the zone of microemulsion at different ratios of surfactant to cosurfactant (e.g., 1 : 1, 2 : 1, and 3 : 1). The effect of formulation variables on different physicochemical characteristics such as dispersibility tests, globule size, and zeta potential was studied and (2) to formulate ACF-Na preconcentrate L-ME loaded solid CA nanocomposite microbeads using SA and Neusilin US2 as carriers and calcium chloride as cross-linking agent. To study the effects of various formulation and process variables. In this research work we investigate the effect of various concentrations of SA, calcium chloride, and Neusilin US2 on mean particle size, actual drug content, drug entrapment efficiency, mechanical strength, and* in vitro* drug release potential of the formulated nanocomposite microbeads.

## 2. Material and Methods

*Materials*. ACF-Na was obtained as a gift sample from Microlabs, Bengaluru, Karnataka, India. SA was gift sample from F.M.C. International biopolymers, Willington, Ireland, to Signet Chemical Corporation Pvt. Ltd., Mumbai, India. Labrafac PG (propylene glycol dicaprylocaprate EP/NF), Labrafil 2125 CS (Linoleoyl macrogolglycerides), and Labrasol (Caprylocaproyl macrogol-8 glycerides EP) are gift samples from Gattefosse groups, Saint Priest Cedex, France. Neusilin US2 (magnesium aluminometasilicate) was gift sample from Fuji Chemical Industry Co., Burlington, NJ, USA, to Gangwal Chemicals Ltd., Mumbai, India. All other reagents and solvents used were of analytical grade satisfying pharmacopoeias specifications.

### 2.1. Preformulation Studies

#### 2.1.1. Saturation Solubility Study

Solubility studies were conducted by placing an excess amount of ACF-Na (approximately 1 g) in a 50 mL volumetric flask containing 10 mL of different oils, surfactants, and cosurfactants (Table 1). Then, the mixture was vortexes for 30 min and was kept for 48 h at room temperature in a water bath by shaking with mechanical shaker to facilitate the solubilisation. The samples were centrifuged at 3000 g for 15 min to remove the undissolved ACF-Na. The supernatant was taken and diluted with methanol for quantification of ACF-Na at 275 nm using Shimadzu 1201 UV-visible spectrophotometer (Shimadzu 1201, Japan) [17].

#### 2.1.2. Fourier Transform Infrared (FT-IR) Spectroscopic Analysis

The drug polymer interactions were studied by using FT-IR spectrophotometer (Shimadzu 1700S). The samples were prepared by adopting KBr pellet technique and scanned from 4000 to 450 cm^−1^ taking air as the reference.

#### 2.1.3. Differential Scanning Calorimetry (DSC)

Differential scanning calorimetry (DSC) was performed using DSC-60 (Shimadzu, Tokyo, Japan) calorimeter to study the thermal behaviours of ACF-Na alone and mixture of ACF-Na and polymer. The instrument comprised calorimeter (DSC-60), flow controller (FCL-60), thermal analyser (TA-60), and operating software (TA-60). The samples were heated in sealed aluminium pans under nitrogen flow (30 mL/min) at a scanning rate of 5°C/min from 24 ± 1 to 250°C. Empty aluminium pan was used as reference. The heat flow as a function of temperature was measured for the drug and drug polymer mixture.

#### 2.1.4. X-Ray Powder Diffractometry (XRPD)

The crystalline nature of ACF-Na and polymers in the manufacturing process was performed by using Philips X-ray powder diffractometry (model; PW 1710) with copper target. The X-ray diffraction patterns of pure drug and the optimized drug loaded formulations were recorded using the radiation at 30 kv and 25 mA^0^, scanning speed at 20/min^−1^, and 40 to 400 diffraction angle (2*θ*) ranges.

#### 2.1.5. Constructing Pseudoternary Phase Diagram

From solubility study, Labrafac PG, Labrasol, and Span 80 were selected as oil, surfactant, and cosurfactant, respectively. The stable microemulsion region was identified by constructing pseudoternary phase diagram containing different proportion of surfactant:cosurfactant (Km value 1 : 1, 2 : 1, and 3 : 1), oil, and water. In brief, the different concentration of surfactant and oil was mixed at ratio of 1 : 9, 2 : 8, 3 : 7, 4 : 6, 5 : 5, 6 : 4, 7 : 3, 8 : 2, and 9 : 1 in preweighed test tube. To the resultant mixtures, double distilled water was added dropwise till the first sign of turbidity in order to identify the end point and after equilibrium. After complete equilibrium was reached, the mixtures were checked visually for transparency and by visualizing through optical polarizer (Nikkon, Japan). The systems which appeared to be black when visualized through the crossed polarizers were deemed to be within the microemulsion region. The amount of each of the components (% w/w) for a particular composition was determined with the help of pseudoternary phase diagram [18].

#### 2.1.6. Preparation of Liquid Microemulsion of ACF-Na

The phase diagrams were constructed at different Km values and which Km values ratio gives high microemulsion region obtained was selected for formulation of L-MES. For 200 mg of ACF-Na and Labrafac PG (10% w/w) was placed in glass vial, warmed on water bath. The mixture of Labrasol and Span 80 in the proportion 2 : 1 (40% w/w) was added into the oily dispersion. Then, the components were mixed gently by using microhomogenizer and vortexes at 37°C until drug was completely dissolved to form clear homogeneous mixture. Then, the mixture was sealed in glass vial and stored at room temperature until use [19].

#### 2.1.7. Preparation of Solid CA-Neusilin US2 Nanocomposite Microbeads

The ACF-Na preconcentrate L-MES was saturated with 20 mL of SA solution (0.5, 1.0, 1.5, and 2% w/v) and by adding Neusilin US2 (0.4, 0.6, 1.0, and 1.5% w/w) as adsorbent stirred 500 rpm for 30 min at 40°C. The bubble-free dispersion was dropped through a 10 mL glass syringe fitted with 24-gauge hypodermic needle into 50 mL (0.5, 2.0, 4.0, and 6.0% w/v) of calcium chloride solution and was stirred at 200 rpm for 30 min. The droplets from the dispersion instantaneously gelled into discrete matrices upon contact with the solution of calcium chloride. After specified stirring time and stirring speed, gelled beads were separated by filtration, washed with isopropyl alcohol and further 3 × 50 mL volumes of deionized water, and finally dried at 60°C for 4 h in a hot air oven. The various concentrations of SA, Neusilin US2, and calcium chloride were evaluated on physicochemical and drug release characteristics of nanocomposite microbeads.

### 2.2. Characterization and Evaluation of L-ME, Solid Nanocomposite Microbeads

#### 2.2.1. Visual Assessment Test (Dilution Effect)

L-ME was diluted with 1-, 25-, 50-, 100-, 150-, and 200-fold with pH 4.5 mixed PBS. The contents were then mixed gently with magnetic stirrer at room temperature. The tendency to form emulsion spontaneously and also the arrangement of emulsion droplets were observed. When droplets spread easily in the medium and formed a fine milky emulsion, this indicates very good tendency. When there was poor or no emulsion formation with immediate coalescence of oil droplets, especially when stirring, being stopped, this indicates less tendency.

#### 2.2.2. Globule Size, Polydispersity Index (PDI), and Zeta Potential

The L-MES was diluted to 10 times with distilled water and globule size, PDI, and zeta potential were determined using Malvern Zetasizer (Nano ZS90) by dynamic light scattering (DLS) by monitoring at 25°C at a scattering angle 173° in which measure size ranges between 6 nm and 0.6 *μ*m. The nanometric size range of the particle was retained even after 100 times dilution with water which proves the compatibility of the system with excess water [20, 21].

#### 2.2.3. Measurement of Micromeritic Properties of Nanocomposite Microbeads

The flow properties were investigated by measuring the angle of repose of drug loaded microbeads using fixed-base cone method [22]. The angle of repose was calculated by using the following formula. Each experiment was carried out in triplicate (*n* = 3). Consider

(1) $$\begin{array}{c} \text{Angle}\;\text{of}\;\text{repose}\left(\theta \right)=\tan^{-1}\left(\frac{h}{r}\right), \end{array}$$

where *h* is cone height and *r* is radius of circular base formed by the microbeads on the ground.

The bulk and tapped densities of the formulated microbeads were evaluated by using the bulk density apparatus. The experiment was repeated in triplicate (*n* = 3). The respective densities were calculated by using the following formulas:

(2) Bulk  densitygm/cc=Mass  of  the  samplegBulk  volumemL.

Compressibility index or Carr's index value of microbeads was computed according to the following equation:

(3) Carr's  index(%) =Tapped  density−Bulk  densityTapped  density×100.

Hausner's ratio of microbeads was determined by comparing the tapped density to the bulk density by using the following equation:

(4) $$\begin{array}{c} \text{Hausner's}\;\text{ratio}=\frac{\text{Tapped}\;\text{density}}{\text{Bulk}\;\text{density}}. \end{array}$$

#### 2.2.4. Mechanical Property of Nanocomposite Microbeads

Mechanical strength of the CA and CA-Neusilin US2 nanocomposite microbeads was carried out using a texture analyser (TA.XT plus, Stable Micro Systems, UK) with a 50 kg load cell equipped with a cylindrical probe of 6 mm in diameter. Large beads were prepared with SA alone and SA/Neusilin US2 dispersion dropped through 1 mL pipette into calcium chloride solution. The fully formed beads were collected, washed with distilled water, and subsequently dried at 60°C for 4 h. One bead was placed on the platform and probe was positioned to touch the bead, recorded as the initial position, and then crosshead speed and probe diameter were set at a constant speed of 1.0 mms^−1^. The probe was removed when the bead was broken or reduced to 50% of its original height [23]. The force and percent displacement were plotted, and the maximum force at 50% displacement, which represents the strength of the beads, was reported.

#### 2.2.5. Particle Size Analysis

The particle sizes of drug loaded CA nanocomposite microbeads were measured by an optical microscope fitted with an ocular and stage micrometer and particle size distribution was calculated. The Olympus model (SZX-12) having resolution of 30 xs was used for this purpose. The instrument was calibrated at 1 unit of eyepiece micrometer which was equal to 1/30 mm (33.33 *μ*m). In all measurements, at least 100 particles in five different fields were examined [24]. Each experiment was carried out in triplicate (*n* = 3).

#### 2.2.6. Scanning Electron Microscopy (SEM) Analysis

The shape and surface characteristics of drug loaded CA nanocomposite microbeads were determined by scanning electron microscopy (model-JSM, 35CF, Jeol, Japan) using gold sputter technique. A working distance of 20 nm, a tilt of zero degree, and accelerating voltage of 15 kv were the operating parameters. Photographs were taken within a range of 50–5000 magnifications.

#### 2.2.7. Drug Entrapment Efficiency (DEE)

Accurately weighed 50 mg of CA nanocomposite microbeads was ground using a mortar and pestle and was dispersed in 100 mL of PBS pH 6.8 ± 0.1. The mixture was sonicated for 30 min and incubated at 37°C for 24 h. Next day, it was stirred for 15 min. The solution was filtered, after suitable dilution, ACF-Na content in the filtrate was analysed at 275 nm using Shimadzu 1201 UV-visible spectrophotometer. The obtained absorbance was plotted on the standard curve to get the exact concentration of the entrapped drug. Calculate the concentration of drug and determine the percentage of actual drug encapsulated in nanocomposite microbeads with dilution factor. The percentage of drug entrapment efficiency was determined using the following relationship:

(5) $$\begin{array}{c} \text{DEE}\left(\%\right)=\left[\frac{\text{Actual}\;\text{drug}\;\text{content}}{\text{Theoretical}\;\text{drug}\;\text{content}}\right]\times 100. \end{array}$$

#### 2.2.8. *In Vitro* Drug Release Studies

The release profiles of ACF-Na from nanocomposite microbeads were examined in three different buffer solutions to mimic various physiological GI-tracts. The media SGF of pH 1.2 was representing the gastric condition; pH 6.8 PBS was a compromise condition between pH of the gastric and small intestine and pH 7.4 is simulated intestinal fluid. The dissolution process was carried out by using USP XIII rotating basket apparatus (Microlabs, Mumbai, India). The drug loaded microbeads (equivalent to 200 mg of ACF-Na) filled in empty capsule shells were put into the basket and rotated at a constant speed at 75 rpm/min and maintained temperature 37°C. The 900 mL SGF of pH1.2 dissolution medium was used in dissolution process for initial 2 h and the test was continued with changing the dissolution media with pH 6.8 PBS up to 6 h and SIF of pH 7.4 at the end of 12 h. At scheduled time intervals, the sample (5 mL) was withdrawn and replaced with the same volume of fresh medium. The withdrawn samples were filtered through a 0.45 *μ*m membrane filter and after appropriate dilution and then estimated for aceclofenac sodium concentration at 275 nm by using UV-visible spectroscopy (Shimadzu 1201, Japan). Finally, corresponding drug content in the samples was calculated from the calibration curve to determine the drug release pattern [25].

#### 2.2.9. Kinetics of Drug Release

In order to understand the mechanism and kinetics of drug release and best fit model for the formulations by using PCP-Disso-V2 software, the drug release data of the* in vitro* dissolution study was analysed with various kinetic equations like zero-order (% release v/s time), first-order (Log % retained v/s time), and Korsmeyer and Peppas equations (*Mt*/*M∞* = *Ktn*). Where *Mt* is the amount of drug released at time *t*, *M∞* is the amount of drug released at infinite time, *K* is the kinetic constant incorporating the structural and geometric characteristics of the beads, and *n* is the diffusional exponent indicative of the release mechanism. Where *n* = 0.5 represents Fickian diffusion, <1.0 represents non-Fickian diffusion, *n* = 1.0 case-II transport, and *n* > 1.0 super case-II transport [26]. Coefficient of correlation (*r*) values were calculated for the linear curves obtained by regression analysis of the above plots.

## 3. Results and Discussions

### 3.1. Preformulation Studies

#### 3.1.1. Solubility Studies

The microemulsion is a clear monophasic liquid formulation consisting of oils, surfactants, cosurfactants, and drug at ambient temperature when introduced to aqueous phase. They have very good solvent properties to solubilize the insoluble solids. The solubility of poor water soluble aceclofenac sodium in various vehicles is presented in Table 1. All the surfactants showed good solubility of the drug. Among all the surfactants tested in this study, Labrasol a medium-length alkyl chain surfactant with HLB 14 was selected as a surfactant because it was reported for its enhanced intestinal absorption of drugs [27]. The solubility of ACF-Na with Labrasol is higher than other surfactants. Moreover, the miscibility of these oils with Labrasol at 2 : 1 volume ratio was investigated by clarity of oil/surfactant mixture. Castor oil, cotton seed oil, sesame oil, peanut oil, and Labrafil 2125 CS were poorly miscible with Labrasol, whereas Labrafac PG was well miscible and formed clear solution. Labrafac PG showed higher drug solubility than other oils. Thus, Labrafac PG was selected as an oily vehicle due to its good solubility and good emulsion forming with surfactant and cosurfactant. Furthermore, Span 80 (HLB 6) was selected as a cosurfactant for its good solubility and for preparing homogenous polymeric dispersion resulting in the improvement of drug loading in the solid microbeads.

#### 3.1.2. FT-IR Spectroscopic Analysis

**Figure 1:**
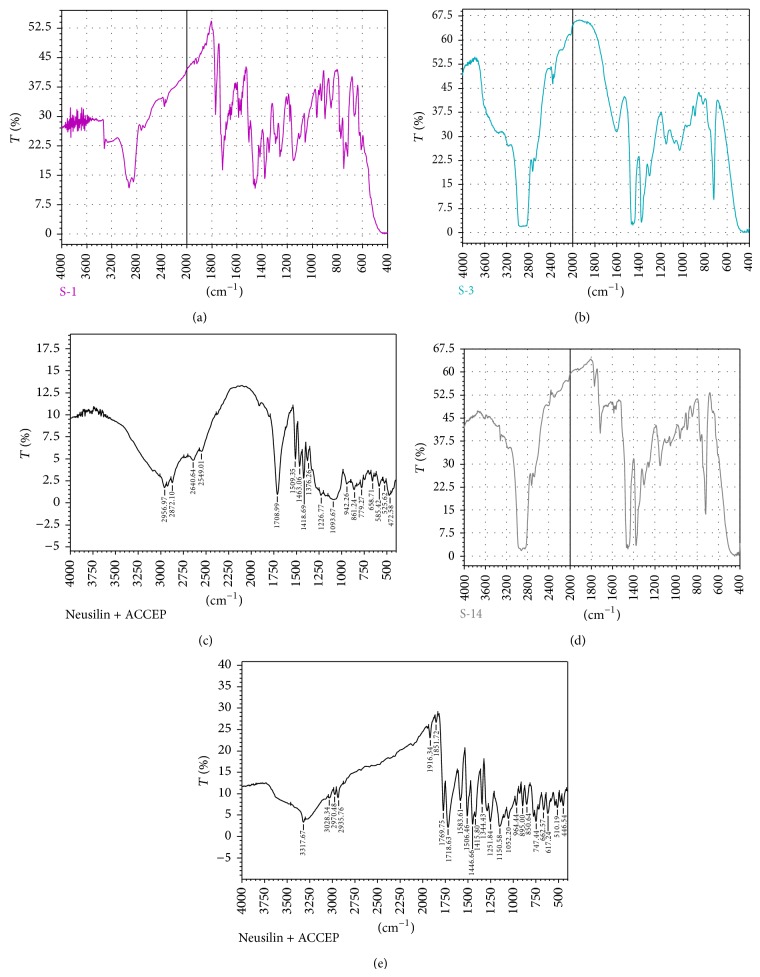
FT-IR spectra: (a) pure aceclofenac sodium, (b) sodium alginate, (c) Neusilin US2, (d) physical mixture of aceclofenac sodium and sodium alginate, and (e) physical mixture of aceclofenac sodium and Neusilin US2.

The molecular interactions of ACF-Na, SA, and Neusilin US2 in the CA nanocomposite microbeads were investigated using FTIR spectroscopy. The characteristic absorption peaks of pure ACF-Na were obtained 3276.5, 2915.5, 1716.5, 1589.3, 1279.6, and 749.4 cm^−1^ corresponding to NH-stretching, C=O stretching of –COO, and –COOH group, respectively (Figure 1(a)). The characteristic absorption peaks SA showed peaks around 3077.15, 2914.98, 1615.34, 1359.60, and 754.05 cm^−1^ reflective of O–H, C–H, COO− (asymmetric), COO− (symmetric), and C–O–C stretching, respectively (Figure 1(b)). The cross-linking process of SA with calcium caused an obvious shift to higher wave numbers and a decrease in the intensity of COO− stretching peaks. Additionally, a change to lower wave numbers and a decrease in the intensity of the C–O–C stretching peak of SA was observed. This indicated the presence of an ionic bond between the calcium ion and the carboxyl groups of SA and partial covalent bonding between the calcium and oxygen atoms of the ether groups [28]. The physical mixture SA and ACF-Na characteristic peaks were obtained at 2820.28, 1591.72, 1384.91, 1102.03, and 766.4 cm^−1^causing a shift in the O–H, COO− (asymmetric), and COO− (symmetric) stretching peaks to lower wave numbers (Figure 1(c)), suggesting that a molecular interaction between SA and ACF-Na was formed due to hydrogen bonding and electrostatic force. The addition of Neusilin US2 in the aceclofenac sodium-loaded CA nanocomposite microbeads caused a change in the carboxylate peaks of SA. The Si–O–Si stretching peak of Neusilin US2 at 1115.24 cm^−1^ (Figure 1(d)) became narrower and moved to a lower wave number 996.15 cm^−1^ (Figure 1(e)), suggesting that Neusilin US2 could interact with SA and ACF-Na in the microbeads. These results suggested no considerable changes in the IR peaks of ACF-Na in the physical mixture thereby indicating the absence of any interactions. Moreover, ACF-Na-Neusilin US2 complex-loaded CA nanocomposite microbeads might have higher drug entrapment efficiency due to being molecularly dispersed in the SA-Neusilin US2 matrix. The peaks shown at 2916.5, 1589.3, 1279.6, and 749.4 cm^−1^ as major peaks for ACF-Na are present in all physical mixtures of drug and polymer which confirms that the drug was molecularly dispersed in the polymer without any interaction.

#### 3.1.3. Differential Scanning Calorimetry (DSC)

**Figure 2:**
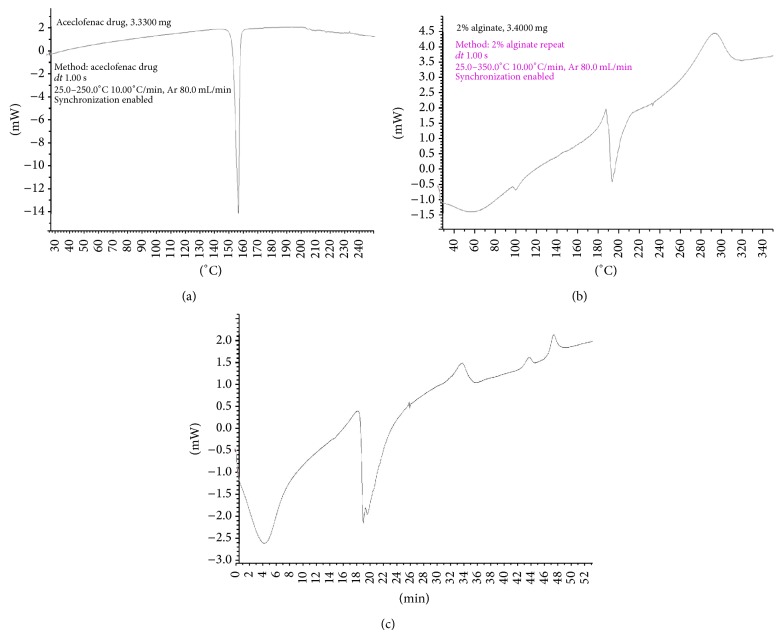
DSC thermograms: (a) pure aceclofenac sodium, (b) CA drug loaded nanocomposite microbeads (AF-3), and (c) CA-Neusilin US2 drug loaded nanocomposite microbeads (AF-7).

DSC is a well-established method often used as a qualitative technique to characterize physical and chemical changes in either enthalpy or heat capacity of a crystalline drug in the polymer matrix during the manufacturing process. The thermal behavior of the pure ACF-Na, drug loaded CA, and CA-Neusilin US2 nanocomposite microbeads were characterized using DSC, as shown in Figure 2. The thermograms of pure ACF-Na showed a sharp endothermic peak at 158.50°C followed by corresponding melting point (Figure 2(a)). However, the DSC thermograms of drug loaded CA-Neusilin US2 microbeads were observed at 189.5°C and 195.6°C, respectively. It can clearly suggest that the CA nanocomposite microbeads containing SA and SA-Neusilin US2 showed an increase in the exothermic peak temperature (Figures 2(b) and 2(c)) and improved the thermal stability. The extra obvious peak of the ACF-Na (158.5°C) was not observed in any type of prepared nanocomposite microbeads and no appreciable change in the melting endothermic peak of the physical mixture as compared to pure ACF-Na. It may indicate that there were no changes in thermal behavior of drug and also the drug was molecularly dispersed in hydrogel matrix.

#### 3.1.4. X-Ray Powder Diffractometry (X-RPD)

**Figure 3:**
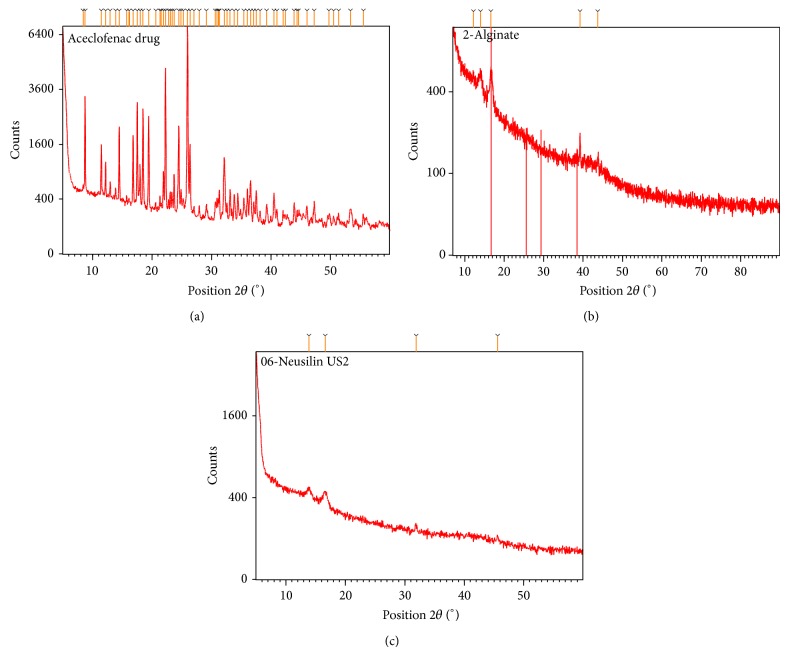
X-Ray diffraction patterns of (a) pure aceclofenac sodium, (b) CA drug loaded nanocomposite microbeads (AF-3), and (c) CA-Neusilin US2 drug loaded nanocomposite microbeads (AF-7).

The X-ray powder diffraction patterns of pure drug are compared with drug loaded CA and CA-Neusilin US2 nanocomposite microbeads. The XRPD scan of plain ACF-Na showed sharp intense peaks of crystallinity (Figure 3(a)), whereas the XRPD pattern of the drug loaded CA and CA-Neusilin US2 nanocomposite microbeads exhibited halo pattern with less intense and denser peaks compared to plain ACF-Na indicating the decrease in crystallinity or partial amorphization of the drug in the microbeads (Figure 3(b)). The intensity of the ACF-Na peaks at 14.47, 24.87, 26.26, 29.95, and 36.55°C (2*θ*), CA microbeads (AF3) at 16.29, 39.23, and 43.76°C (2*θ*), and CA-Neusilin US2 nanocomposite microbeads (AF7) shown at 14.06, 26.65, and 55.65°C (2*θ*) was calculated using D8 TOOLS software. The calculated percentage relative crystallinity value for pure ACF-Na was 92.40% and those of formulations containing SA (AF-3) and SA-Neusilin US2 (AF-7) were 89.25 and 80.09, respectively (Figures 3(b) and 3(c)). This is possibly due to the decrease in the degree of crystallinity of the drug following dispersal in the polymer matrix.

#### 3.1.5. Pseudoternary Phase Diagram

**Figure 4:**
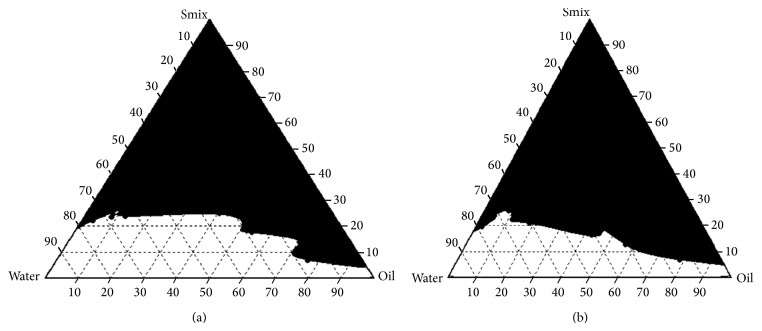
Pseudoternary phase diagram: (a) Km value 1 : 1 and (b) Km value 2 : 1.

This is the first stage to determine the ratio of surfactant:cosurfactant to formulation development of spontaneous microemulsion formation. The boundaries of the microemulsion domains were determined by plotting pseudoternary phase diagrams for the selected components based on solubility studies. Nine different potential combinations of surfactant and cosurfactant mixture to oil at different Km values (1 : 1, 2 : 1) were used for construction of pseudoternary phase diagram. The shaded area in phase diagram shows a stable microemulsion region (Figures 4(a) and 4(b)). The nature of phase behavior of the system was proven to determine concentration of oil, surfactant:cosurfactant and water. Phase diagram at Km value 2 : 1 showed maximum microemulsion region.

### 3.2. Evaluation of Liquid Microemulsion System

#### 3.2.1. Globule Size, PDI, and Zeta Potential

**Figure 5:**
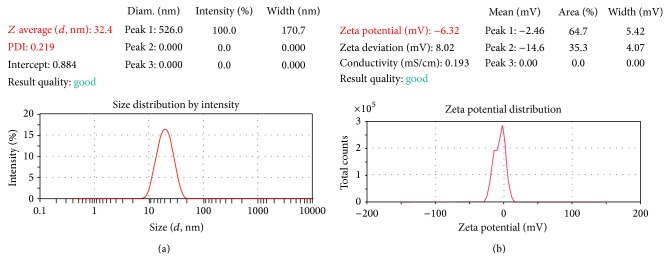
(a) Globule size and PDI of aceclofenac sodium liquid microemulsion system. (b) Globule size and PDI of ACF-Na and SA polymeric dispersion.

The efficiency of L-ME could be estimated by determining the rate of emulsification and droplet size distribution. The droplet size is a crucial factor in microemulsification performance because it determines the rate and extent of drug release as well as absorption [29]. Globule size was found to be 32.4 nm with polydispersity index 0.219 and zeta potential was found to be −6.32 mV (Figures 5(a) and 5(b)). An increase in the ratio of the oil phase (Labrafac PG) resulted in a proportional increase in droplet size. It is well known that the addition of surfactants to the L-ME causes the interfacial film to stabilize and condense, while the addition of cosurfactant causes the film to expand; thus, the relative proportion of surfactant to cosurfactant has varying effects on the droplet size. The decrease in surfactant concentration leads to decreased viscosity of the formulation.

It was observed that increasing the surfactant and cosurfactant ratio decreased the average diameter of oily droplets in the microemulsion. On the other hand, increasing the concentration of oil (Labrafac PG) of the average diameter of oily droplets slightly increased which influences the formation of large size microbeads. Moreover, it was observed that the spontaneous emulsion formation was not efficient with less than 2 : 1 concentration of surfactant : cosurfactant in L-MES. Hence, optimized formulation of preconcentrated L-ME contained Labrafac PG (10%), Labrasol: Span 80 (40%), and water (50%) which are used for the formulation of CA nanocomposite solid microbeads.

### 3.3. Evaluation of CA-Neusilin US2 Nanocomposite Microbeads

#### 3.3.1. Micrometric Properties of Drug Loaded Microbeads

The rheological parameters like angle of repose, bulk density, and tapped density of all microbeads confirm better flow and good packaging properties. All the formulations showed excellent flowability represented in terms of angle of repose (<40°) 20 and compressibility index (<30) and Hausner ratio (<1.24). However, higher calcium chloride concentration in the curing medium influenced the formation of smaller beads because of shrinkage and formation of more compact matrix between SA/Neusilin US2 showed an increased angle of repose (Table 2). Bulk and tapped density of microbeads with Neusilin US2 showed good acceptable range indicating that they have good packability. Moreover, the density of the microbeads increases with increasing the concentration of SA/Neusilin US2 due to formation of more compact and less porosity microbeads than those prepared by SA alone (Table 2).

#### 3.3.2. Scanning Electron Microscopy (SEM) Analysis

**Figure 6:**
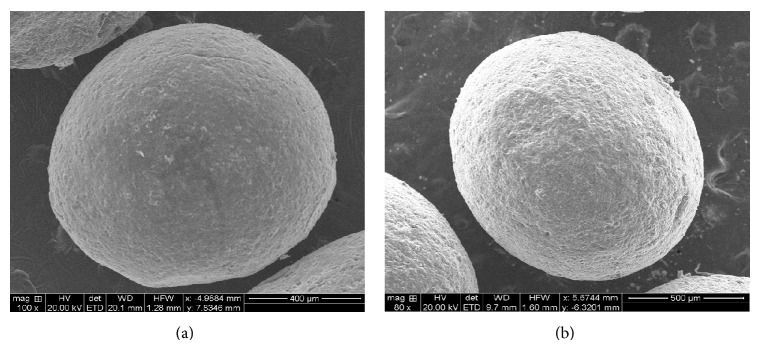
SEM photographs magnifications 200x at 10 kv (a) CA drug loaded nanocomposite microbeads (AF-3) and (b) CA-Neusilin US2 drug loaded nanocomposite microbeads (AF-7).

The SEM photomicrographs of the dried drug loaded CA nanocomposite microbeads are shown (Figure 6). Morphology of the drug loaded CA microbeads was discrete and spherical in shape with a rough outer surface and visible large wrinkles and pores having a sandy appearance due to less compactness (Figure 6(a)). In case of CA-Neusilin US2 nanocomposite microbeads are more spherical; absence of any pores, wrinkles, and also the drug is completely entrapped inside of the polymeric network due to interlocking of Neusilin US2 filler with SA gel (Figure 6(b)).

#### 3.3.3. Mechanical Property of Microbeads

**Figure 7:**
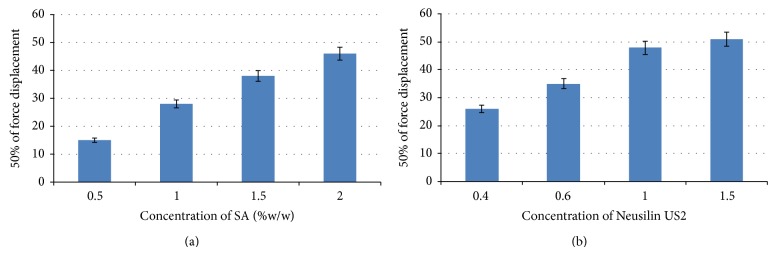
(a) Effect of SA on mechanical strength of CA nanocomposite microbeads. (b) Effect of Neusilin on mechanical strength of CA nanocomposite microbeads.

The maximum force for 50% displacement or breakdown was used to evaluate the strength of the microbeads. The mechanical strength of the CA nanocomposite microbeads was increased by increasing the concentration of SA in the formulation (Figure 7(a)). The maximum force for 50% displacement of the microbeads gradually increased with increasing concentration of Neusilin US2 content. The content of Neusilin US2 above 1.5% w/w did not show any greater changes in the strength of the beads due to complete saturation level of Neusilin US2 with SA. This result indicated that the Neusilin US2 interacts with SA adsorbing oil and water content in interior level of the beads during the cross-linking process and could create a dense matrix structure that reinforced the strength of the microbeads (Figure 7(b)).

#### 3.3.4. Particle Size Analysis

The mean particle sizes of drug loaded microbeads were performed by optical microscopy, and mean particle sizes of the CA microbeads (AF1–AF12) were obtained in the range between 386 ± 1.22 and 667 ± 0.55 (Table 2). The results indicated that the proportional increase of SA in the formulations increases the mean particle size of microbeads. This could be attributed to an increase in relative viscosity at higher concentration of SA and formation of large droplets during addition of polymer solution to the curing medium. On the other hand, while increasing the concentration of Neusilin US2, the mean particle size significantly decreases. This may be due high water adsorption capacity of Neusilin US2 and formation of compact dense matrix with SA. Further, the concentration of calcium chloride increases in the curing medium would significantly decrease the mean particle size of nanocomposite microbeads (Table 2). It has been stated that when a drop of alginate solution comes in contact with calcium ions, gelation occurs instantaneously. As Ca^+2^ ions penetrate into interior part of droplets, water is squeezed out of the interior part of droplets resulting in contraction of microbeads [30]. The size of the spherical matrix could easily be controlled by varying the stirring speed and cross-linking time of the system (results are not reported).

#### 3.3.5. Drug Entrapment Efficiency (DEE)

As shown in Table 3, actual drug concentration in the microbeads was evaluated and was found to be in the range 29.56 ± 0.33 to 49.75 ± 0.25 mg/50 mg. The SA concentration increases; consequently, the actual drug loading is high due to increase in hydrophobicity leading to better adsorption of the microemulsion at the boundary phase of the droplets [AF-1 to AF-4]. When the concentration of Neusilin US2 increases the actual drug content predominately increases [AF-5 to AF-8] because of less leakage of drug from microbeads during the curing period with constant stirring speed (1500 rpm) due to the formation of dense matrix with SA and Neusilin US2. The effect of various process and formulation parameters on the drug entrapment efficiency of microbeads was investigated, by increasing the concentration of SA [AF-1 to AF-4]; the percentage of drug entrapment efficiencies was found to be in the range 59.12 ± 0.35 to 93.30 ± 0.55 w/w. It was observed that the drug entrapment efficiencies increased progressively with increasing the concentration of SA resulting in the formation of larger beads entrapping the greater amount of the drug. On other hand, the addition of Neusilin US2 drug entrapment efficiency of the CA nanocomposite microbeads is obtained in the range 91.90 ± 0.25 to 99.50 ± 0.05 [AF-5 to AF-8]. When the concentration of Neusilin US2 increases in the SA dispersions the percentage of drug entrapment efficiency progressively increases. The formation of hydrogen bonds between Neusilin US2 and SA to create numerous points of contact could produce a loose three-dimensional gel like structure throughout the composite dispersion. The higher the concentration of Neusilin US2, the greater the number of contact points in the dispersion. These interactions could result in an enhancement of the viscosity and thixotropic properties of the composite dispersions, more amount of drug adsorbed onto the Neusilin US2, increased the barrier to preventing the leaking of drug from the CA microbeads in the curing medium during the manufacturing process.

Increasing calcium chloride concentration, the percentage of drug entrapment efficiencies was found to be in the decreasing range 94.60 ± 0.69 to 89.20 ± 1.07 (Table 3). From the results, it is obvious that increasing calcium chloride concentration in the curing medium produced microbeads with higher levels of Ca^2+^ ions possibly due to shrinkage occurring during gelation period. This shrinkage could have led to a shorter path length for drug leakage and, therefore, higher drug loss [31]. On other hand, further increase in the concentration of calcium chloride above 6% w/w did not change the drug loading of microbeads (results are not reported). This could be due to possible saturation of calcium binding sites in the guluronic acid chain, preventing further Ca^2+^ ions entrapment and, hence, cross-linking was not altered with higher concentration of calcium chloride solution.

#### 3.3.6. *In Vitro* Drug Release Studies

**Figure 8:**
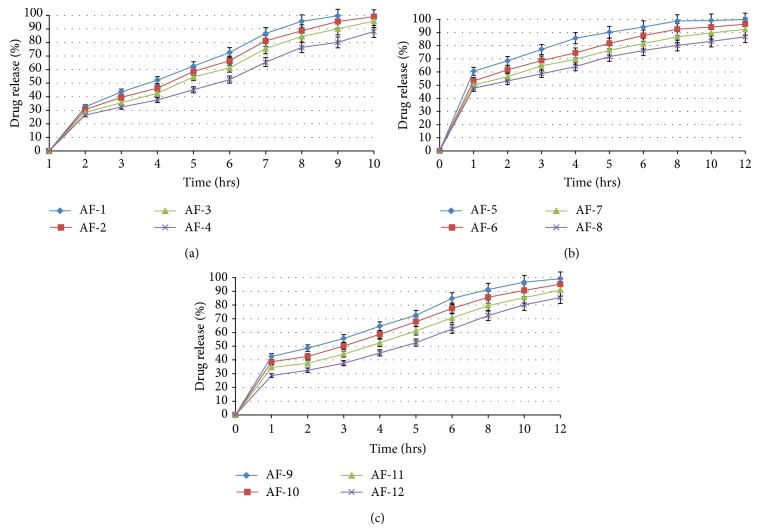
(a) Effect of SA on cummulative percentage of drug release from CA nanocomposite microbeads. (b) Effect of Neusilin US2 on cummulative percentage of drug release from CA nanocomposite microbeads. (c) Effect of calcium chloride on cummulative percentage of drug release from CA-Neusilin US2 nanocomposite microbeads.

ACF-Na has been slightly soluble in water and showed very poor solubility in the acidic buffer media. However, the percentage of drug release from CA nanocomposite microbeads [AF-1 to AF-4] in SGF pH 1.2 within 2 h in the range of 32.54, 30.56, 28.35, and 26.55 w/w may be due to the stability of alginate at lower pHs and conversion of calcium alginate to the insoluble alginic acid to form tightening of the gel mesh work. On the other hand, the percentage of drug release in pH 6.8 PBS at 6 h is shown in the range of 52.33, 49.52, 44.25, and 37.65 w/w. Further, the percentage of drug release from microbeads in pH 7.4 SIF up to 12 h is 99.65, 97.12, 95.45, and 89.12 w/w (Figure 8(a)). As the concentration of SA increased, release rate of ACF-Na from the nanocomposite microbeads decreased, which shows biphasic pattern; that is, an initial rapid release (burst effect) phase was followed by a second slower drug release phase. The first phase might be disintegration of alginate in the presence surfactant and cosurfactant mainly based on drug diffusion through the small pores and cracks. The second phase exhibited slow release pattern, which was accompanied by diffusivity of polymer relaxation due to change in pH of the medium. The SA concentration in the formulation greatly influenced the steady state release of drug from the nanocomposite microbeads.

The percentage of drug release from ACF-Na from CA-Neusilin US2 nanocomposite microbeads [AF-5 to AF-8] in SGF of pH 1.2 within 2 h was obtained in the range of 60.56 to 47.65 w/w. The results suggested that the presence of silanols on its surface makes it a potential proton donor as well as a proton acceptor, which increases the initial drug release by lower concentration of Neusilin US2 in acidic medium. The release rate during 6 h in pH 6.8 PBS and SIF of pH 7.4 after 6 h–12 h was decreased from 94.15 to 76.25 and from 98.96 to 86.75 w/w (Figure 8(b)). The observed results suggested that the hydrogen bonding between silanols and drug as well as interaction between the drug and metal ions on the surface of Neusilin US2 was stabilizing mechanism of ACF-Na in its amorphous state and increases the aqueous solubility at alkaline medium; due to this reason the maximum of drug releases in pH 6.8 PBS followed by sustaining SIF of pH 7.4 by electrostatic interaction between surface of the silicate layers of Neusilin US2 with negative charges of carboxylic groups of SA forms thick gel network which acts as a barrier to take prolonged time to diffuse the drug into bulk of the dissolution media.

The effect of cross-linking agent in the curing medium [AF-9 to AF-12] on percentage of drug release rate from CA-Neusilin composite microbeads in SGF of pH 1.2 was observed in range of 42.52 to 28.68 w/w. Further, observed the maximum amount of drug releases during at 6 h in pH 6.8 PBS is shown in the range 84.75, 77.55, 70.55, 62.54 and 99.12, 95.12, 90.75, 85.42 w/w at the end of 12 h in SIF of pH 7.4 (Figure 8(c)). The results indicate that rate and extent of drug release decreased with increase of concentration of calcium chloride, because sodium alginate as a linear copolymer consisting of *β* (1 → 4) mannuronic acid and *α* (1 → 4) L-guluronic acid residues; a tight junction is formed between the residues of alginate with calcium ions [32]. However, in case of higher calcium chloride concentration, the release of the drug from the beads decreased due to increased surface roughness and porosity and also poor entry of dissolution medium into the CA/Neusilin US2 nanocomposite microbeads.

#### 3.3.7. Mechanism of Various Kinetic Models on Drug Release

The* in vitro* dissolution data were analyzed by different kinetic models in order to find out the *n*-value, which describes the drug release mechanism (Table 4). The values of correlation (*r*) were calculated and were found to be more linear for first-order release as compared to zero-order release. Cumulative % drug release was analyzed using PCP Disso-v2 08 software. The kinetic data was best fitted to Korsmeyer and Peppa's model and good regression coefficient was observed. The drug release from microbeads containing SA alone [AF-1 to AF-4] was followed by zero-order but in case of CA-Neusilin US2 nanocomposite microbeads [AF-5 to AF-8] continued to Korsmeyer and Peppa's model. The values of diffusion coefficient ranged between *n* = 0.72480 and 0.8788 indicating the drug release from the microbeads followed by case-II transport controlled by diffusion of CA-Neusilin US2 matrices [33].

## 4. Conclusion

From the investigation results, it can be concluded that the proper selection of optimized formulation conditions is very important to achieve high encapsulation efficiency and to control the release of ACF-Na from the CA-Neusilin US2 nanocomposite microbeads. As mentioned above, numerous studies have confirmed that microemulsion substantially improved solubility/dissolution, absorption, and bioavailability of poorly water soluble drugs. Hence, it was concluded that solid CA-Neusilin US2 nanocomposite microbeads can be efficiently formulated by microemulsification internal gelation technique. The SA and Neusilin US2 as solid fillers were used to convert L-ME preconcentrate into solid nanocomposite microbeads which could enhance drug entrapment efficiency, mechanical strength, and thermal stability, reduce initial burst release, and modulate drug release in both acidic and alkaline environments of gastrointestinal tract for water insoluble drug ACF-Na.

## Figures and Tables

**Table 1:** Saturation solubility of ACF-Na in different vehicles.

Vehicles	Solubility of ACF-Na (mg/mL)
Surfactants	
Tween 20	>1000 mg
Tween 80	>1000 mg
Labrasol	>1000 mg
Oils	
Castor oil	095.22 ± 08.66
Cotton seed oil	112.65 ± 43.03
Sesame oil	136.42 ± 33.04
Peanut oil	148.25 ± 22.17
Labrafil M 1944 CS	192.78 ± 44.15
Labrafac PG	308.12 ± 12.08

Data are the mean ± S.D. *n* = 3.

**Table 2:** Micromeritic properties ACF-Na loaded nanocomposite microbeads.

Batch code	Mean particle size	Angle of repose	Bulk density	Tapped density	Carr's index	Hausner's ratio
	[*μ*m]	[*θ*]	[gm/mL]	[gm/mL]	[ci]	
AF-1	386 ± 1.22	31.21 ± 0.21	0.469 ± 0.95	0.585 ± 0.65	19.69	1.24
AF-2	552 ± 0.85	26.10 ± 0.37	0.556 ± 0.34	0.658 ± 0.64	15.50	1.18
AF-3	586 ± 1.02	20.80 ± 0.12	0.596 ± 0.38	0.688 ± 0.12	14.62	1.15
AF-4	667 ± 0.55	20.10 ± 0.32	0.602 ± 0.15	0.690 ± 0.33	14.45	1.14
AF-5	572 ± 0.80	23.30 ± 0.10	0.626 ± 0.64	0.756 ± 0.55	17.19	1.20
AF-6	568 ± 0.75	22.65 ± 0.20	0.597 ± 0.12	0.705 ± 0.85	15.32	1.18
AF-7	502 ± 0.45	19.65 ± 0.25	0.588 ± 0.62	0.685 ± 0.87	15.03	1.16
AF-8	496 ± 0.38	18.75 ± 0.62	0.562 ± 0.30	0.647 ± 0.54	13.29	1.15
AF-9	565 ± 0.22	25.45 ± 0.45	0.557 ± 0.42	0.678 ± 0.22	17.84	1.22
AF-10	536 ± 0.12	22.75 ± 0.82	0.545 ± 0.18	0.656 ± 0.12	16.92	1.20
AF-11	501 ± 0.15	20.15 ± 0.55	0.539 ± 0.23	0.638 ± 0.34	15.52	1.18
AF-12	488 ± 0.35	19.32 ± 0.22	0.529 ± 0.31	0.618 ± 0.58	14.45	1.16

Data are the mean ± S.D. *n* = 3.

**Table 3:** Composition and physical characteristics of drug loaded nanocomposite microbeads.

Formulation code	S. A	Neusilin US2	Calcium chloride	Actual drug content	DEE
	(%w/w)	(%w/w)	(%w/v)	(mg/50 mg)	(%w/w)
AF-1	0.5	—	4	29.56 ± 0.33	59.12 ± 0.35
AF-2	1.0	—	4	37.65 ± 0.15	75.30 ± 0.56
AF-3	1.5	—	4	44.85 ± 0.28	89.70 ± 0.36
AF-4	2.0	—	4	46.65 ± 0.62	93.30 ± 0.55
AF-5	1.5	0.4	4	45.95 ± 0.12	91.90 ± 0.25
AF-6	1.5	0.6	4	47.45 ± 0.55	94.90 ± 0.55
AF-7	1.5	1.0	4	49.25 ± 0.32	98.50 ± 0.09
AF-8	1.5	1.5	4	49.75 ± 0.25	99.50 ± 0.05
AF-9	1.5	1.0	0.5	47.30 ± 0.87	94.60 ± 0.69
AF-10	1.5	1.0	2	46.80 ± 0.74	93.60 ± 1.14
AF-11	1.5	1.0	4	45.40 ± 0.14	91.82 ± 1.04
AF-12	1.5	1.0	6	44.60 ± 0.42	89.20 ± 1.07

All the formulations contain 200 mg of aceclofenac sodium. Data are expressed ± S.D. *n* = 3.

**Table 4:** Effect of various kinetic models on drug release.

Formulation code	Effect of various kinetic models on drug release					
	Zero-Order	First-Order	Higuchi matrix	Korsmeyer-Peppas	Korsmeyer-Peppas	
					*n*-values	*k*-values
F1	0.9117	0.9114	0.9124	0.9762	0.7248	2.7889
F2	0.9209	0.9174	0.9113	0.9801	0.7456	2.6783
F3	0.9392	0.9221	0.9108	0.9840	0.7515	2.4240
F4	0.9474	0.9329	0.9103	0.9950	0.7711	2.0693
F5	0.9521	0.9830	0.9097	0.9257	0.7764	1.9066
F6	0.9399	0.9803	0.9082	0.9291	0.6535	2.0130
F7	0.9372	0.9811	0.9088	0.9262	0.5449	2.1643
F8	0.9278	0.9801	0.9076	0.9217	0.5523	2.2625
F9	0.9129	0.9475	0.9702	0.9148	0.6482	2.4075
F10	0.9007	0.9568	0.9720	0.9115	0.7361	2.6019
F11	0.9826	0.9624	0.9842	0.9162	0.8704	2.8220
F12	0.9846	0.9700	0.9868	0.9153	0.8788	2.6173

All the results show S.D. *n* = 3; *n*: diffusion exponent (slope) related to mechanism of drug release, according to equation Mt/*M* = *K* ^*tn*^, and *r*: regression coefficient.
